# Animal Models of Non-Respiratory, Post-Acute Sequelae of COVID-19

**DOI:** 10.3390/v17010098

**Published:** 2025-01-14

**Authors:** Abigail Vanderheiden, Michael S. Diamond

**Affiliations:** 1 Department of Medicine, Washington University School of Medicine, St. Louis, MO 63110, USA; abigailv@wustl.edu; 2 Department of Molecular Microbiology, Washington University School of Medicine, St. Louis, MO 63110, USA; 3 Department of Pathology & Immunology, Washington University School of Medicine, St. Louis, MO 63110, USA; 4 The Andrew M. and Jane M. Bursky Center for Human Immunology and Immunotherapy Programs, Washington University School of Medicine, St. Louis, MO 63110, USA; 5 Center for Vaccines and Immunity to Microbial Pathogens, Washington University School of Medicine, St. Louis, MO 63110, USA

**Keywords:** coronavirus, long COVID, pathogenesis, mouse models, brain, heart, gastrointestinal tract

## Abstract

Post-acute sequelae of COVID-19 (PASC) are a diverse set of symptoms and syndromes driven by dysfunction of multiple organ systems that can persist for years and negatively impact the quality of life for millions of individuals. We currently lack specific therapeutics for patients with PASC, due in part to an incomplete understanding of its pathogenesis, especially for non-pulmonary sequelae. Here, we discuss three animal models that have been utilized to investigate PASC: non-human primates (NHPs), hamsters, and mice. We focus on neurological, gastrointestinal, and cardiovascular PASC and highlight advances in mechanistic insight that have been made using these animal models, as well as discussing the sequelae that warrant continued and intensive research.

## 1. Introduction

In 2019, a novel betacoronavirus, SARS-CoV-2, emerged and rapidly caused a pandemic of respiratory disease termed coronavirus disease of 2019 (COVID-19). Symptoms of acute COVID-19 include cough, fever, sore throat, nasal congestion, loss of the sense of smell, headache, nausea, and shortness of breath. Despite the resolution of the acute respiratory tract infection, many patients develop post-acute sequelae of COVID-19 (PASC), also known as long COVID, which is defined as symptoms that persist for at least 3 months after initial infection, but can continue for years. Current estimates indicate that between 5% and 10% of non-hospitalized COVID-19 patients develop PASC, with these rates increasing to almost 50% in hospitalized patients [1,2,3,4,5]. The risk of developing PASC increases with initial disease severity, female sex, and with pre-existing conditions including obesity, chronic lung disease, diabetes, or hypertension [6,7]. The only factor known to decrease the risk of developing PASC is prior vaccination [8,9]. PASC are heterogenous and involve many organ systems, including the cardiovascular system (chest pain and heart palpitations), the respiratory system (cough and shortness of breath), the gastrointestinal tract (abdominal pain, nausea, and diarrhea), the pancreas (increased risk of diabetes), the central nervous system (cognitive impairment, fatigue, and memory loss), the musculoskeletal system (muscle weakness and exercise intolerance), and the kidneys, spleen, and liver (reviewed in [2]). There are no approved treatments for PASC, and the persistence of symptoms contributes to a decline in quality of life [10]. Given the numbers of people affected, an enhanced understanding of the etiology and pathogenesis of PASC is an urgent public health problem.

The heterogeneous nature of PASC and the diverse organ systems involved render it challenging to elucidate underlying and unifying mechanisms. Most studies of patients focus on one organ system, which has led to at least four disparate hypotheses as to the etiology of PASC. One hypothesis is that PASC is driven by persistence of viral antigens and infection in some organs of the body (e.g., gastrointestinal (GI) tract or lung) that drive continued inflammation [11,12,13]. How SARS-CoV-2 antigen persists without active viral replication or clearance by the immune system remains unclear. A second hypothesis proposes that the initial, acute infection causes a “mis-programming” of the immune system, which results in either autoimmunity and/or dysregulated immunity that promotes persistent inflammation [14,15,16,17,18]. A third hypothesis postulates that acute or persistent SARS-CoV-2 infection in the GI tract causes long-term disruption to the microbiota, which results in altered metabolism, immunity, and homeostasis [19,20,21,22,23]. A fourth hypothesis proposes that persistent coagulation abnormalities observed in some PASC patients are driven by the ability of the spike protein of SARS-CoV-2 to bind fibrinogen and promote fibrin clot formation, resulting in endothelial dysfunction and multi-organ symptoms [24,25,26,27]. While longitudinal studies of PASC patients have identified some features that support these hypotheses, determining which is most accurate and the underlying basis for its pathogenesis will require further study in humans and relevant animal models.

In this review, we describe the currently available animal models for different features of PASC. As an excellent review was written recently on animal models of respiratory PASC [28], we focus here on extrapulmonary organ systems (neurological, gastrointestinal, and cardiovascular) impacted by PASC and recent developments in animal models. We discuss three major animal models: non-human primates (NHPs), Syrian golden hamsters, and mice. We compare how different animal models recapitulate specific features of PASC and highlight advances that they have provided in our understanding of the underlying disease. Lastly, we discuss symptoms and organ systems of PASC that entirely lack or require better animal models.

## 2. Neurological PASC

Neurological sequelae, including memory problems, attention and concentration difficulties (so-called brain fog), anosmia, and headache, are some of the most frequently reported symptoms in PASC patients [4,8,29,30]. Cognitive testing of PASC patients demonstrated impaired declarative and working memory for at least one year post-infection [31,32], and imaging studies showed decreases in brain gray matter volume, evidence of multi-focal brain hyperintensities, and blood–brain barrier (BBB) disruption [32,33,34,35]. Thus, self-reported neurological symptoms in PASC patients correlate with measurable differences in cognitive capacity and brain structure. While small amounts of viral RNA or antigens have been detected sporadically near blood vessels in the brains of patients that died from COVID-19, in the majority of cases, infection of the central nervous system (CNS) by SARS-CoV-2 did not occur (reviewed in [36]). Despite the lack of infectious virus in the CNS, autopsy studies of the brains of COVID-19 patients observed microgliosis, astrogliosis, decreased neurogenesis, BBB disruption, increased levels of inflammatory cytokines, T cell infiltration, and alterations in synaptic protein levels [34,35,36,37,38,39,40,41,42]. Addressing how and why an acute respiratory virus causes persistent neurological dysfunction, neuroinflammation, and cognitive changes is key to understanding neurological PASC. A main challenge in studying the neurological effects of COVID-19 (neuroCOVID) has been to identify animal models that have robust infection of the respiratory tract, persistent neurological pathology, but little-to-no productive infection in the CNS.

***(a) NHP models of neuroCOVID.*** Soon after the emergence of SARS-CoV-2, NHPs (Rhesus or Cynomolgus macaques) were established as models for the pulmonary characteristics of COVID-19. Intranasal and intratracheal infection with SARS-CoV-2 resulted in productive infection of the respiratory tract and lung pathology [43]. However, the utility of NHPs as a model of neuroCOVID is less clear. Some studies detected sparse, if any, viral protein/RNA in the brain (primarily near blood vessels), whereas others reported infection of neurons in the olfactory bulb, after intranasal and intratracheal inoculation [44,45,46,47]. These conflicting results may be due to differences in the SARS-CoV-2 variants used, the sample collection timepoint, the viral detection method, or the cutoffs chosen to define positive viral signals. Regardless, neuroinvasion through the olfactory tract does not occur in humans, as olfactory sensory neurons are not permissive to SARS-CoV-2 infection [48]. Notwithstanding this point, with or without infectious SARS-CoV-2, viral antigens, or RNA in the CNS, several NHP studies reported evidence of microgliosis, astrogliosis, infiltrating T cells, microhemorrhages, and Lewy body formation in the brains of SARS-CoV-2-infected animals extending to at least 5 weeks after recovery from acute infection [44,47,49]. Thus, NHP models recapitulate at least some of the neuroinflammation and pathology in the CNS observed in humans with neurological sequelae of COVID-19. To date, this neuroinflammation and viral RNA in the NHP brain have not been linked to changes in behavior after SARS-CoV-2 infection.

***(b) Hamster models of neuroCOVID.*** Syrian golden hamsters are an excellent model for COVID-19-associated lung disease. They are readily infected via intranasal inoculation by almost all variants of SARS-CoV-2 and develop severe lung inflammation and disease [50,51]. Investigation into the utility of hamsters as a neuroCOVID model showed that viral RNA can be detected at acute timepoints (2–5 days post-infection) in the olfactory bulb (OB); however, levels are low and there is conflicting evidence as to whether infectious virus is present. These disparities may be due to differences in methodological approaches to viral detection or the SARS-CoV-2 variant used for infection. Additionally, viral RNA has not been consistently detected in other regions of the brain [52,53,54,55]. Thus, intranasal infection of hamsters does not result in widespread infection of the CNS, but may cause limited and transient infection of the OB. SARS-CoV-2 RNA in the OB was associated with the induction of cytokines, chemokines, and interferon (IFN)-stimulated genes at acute timepoints [52,53,54]. While viral RNA in the OB was not detected past 8–10 days post-infection, expression of type I and III IFN-stimulated genes, chemokines (CXCL10, CCL5), and pro-inflammatory cytokines (IL-1β, IL-6) was sustained at least through 30 days post-infection [53,55,56]. Thus, SARS-CoV-2 infection of hamsters results in low levels of viral RNA at acute timepoints and a persistent antiviral immune response in the OB.

Efforts have been made to investigate the cellular immune response in the brain in hamsters infected with SARS-CoV-2. Multiple studies have demonstrated acute and persistent microgliosis in the OB, as well as the cortex, cerebellum, brain stem, amygdala, and hippocampus, after SARS-CoV-2 infection [37,53,54,55,57,58]. Activated microglia likely contribute to cytokine expression in the brain, as they were shown to be a source of elevated IL-1β levels at acute timepoints in the hippocampus and brain stem [37]. How microglia become activated in areas of the brain lacking viral RNA remains unclear, but one hypothesis is that BBB disruption could trigger microgliosis. In support of this idea, hamsters infected with SARS-CoV-2 had increased numbers of string vessels (remnants of capillaries without blood flow) at acute timepoints, which are indicators of vascular dysfunction [59]. Additionally, areas of the brain with microgliosis showed signs of BBB disruption as measured by leakage of fibrinogen into the parenchyma at acute timepoints [32]. Although astrocytes can also participate in immune responses in the brain, only one group to date has found astrogliosis in the hippocampus and medulla of hamsters [37]. Thus, after SARS-CoV-2 infection, hamsters show evidence of microglial activation in many regions of the brain. Further studies are needed to define the drivers of persistent microglial activation, the impact that microglial activation has on neuronal processes, and the potential roles of other glial cells, including astrocytes and oligodendrocytes.

Hamster studies have begun to provide insight as to how neuroinflammation associated with SARS-CoV-2 infection impacts neuronal function and behavior. Microscopy studies showed decreases in the total number of neurons in the hippocampus, cortex, and amygdala between 14 and 21 days post-infection [57,58]. Further analysis of the hippocampus demonstrated a decline in the number of neuroblasts, the progenitor cell of neurons, at acute timepoints, which could contribute to a decrease in mature neurons at subacute timepoints (1–2 months later) [37]. Cortical neurons had increases in hyperphosphorylated Tau and alpha-synuclein proteins at 14 days post-infection, which suggests changes in neuronal health [57]. Transcriptional profiling confirmed that neuronal metabolism is perturbed after SARS-CoV-2 infection in the striatum, cerebellum, and OB [53,60]. In the OB, dysregulation of genes associated with synaptic signaling and neuronal plasticity were detected [53]. Neuronal changes in the OB were linked to anosmia using a buried-food test at 3 to 7 days post-infection, but persistent anosmia was not observed [52,53,56,61]. However, sustained changes in pain tolerance were observed in the hamster: SARS-CoV-2 RNA was present in the dorsal root ganglion, which promoted neuropathy, resulting in a lower pain tolerance in a test of mechanical hypersensitivity at 28 days post-infection [62]. Despite providing some insight into our understanding of neurological sequelae of PASC, more studies in the hamster are needed to link how viral RNA and neuroinflammation promote persistent alternations in neuronal function.

***(c) Mouse models of neuroCOVID*.** The SARS-CoV-2 strain that emerged from China in 2019 did not bind efficiently to the mouse ACE2 receptor, making initial murine infection studies difficult. Several different strategies were used to generate murine COVID-19 models including: (1) Serial passage and/or mutagenesis of the virus to enhance binding to mouse ACE2, resulting in mouse-adapted SARS-CoV-2 strains [63,64]. One strain in particular, MA10, is utilized widely due to its ability to cause acute and persistent lung disease [64,65]. While low amounts of viral RNA have been detected in the CNS by some groups, infectious MA10 virus has not been recovered [64,66,67]. (2) Transgenic mice expressing human ACE2 under an epithelial cell promoter (K18) can be infected by most SARS-CoV-2 strains and develop severe, often lethal, disease [68,69,70]. While K18-hACE2 mice have been an important model for studying severe lung disease and testing countermeasures, they may be less useful for investigating neuroCOVID or other extrapulmonary pathogenesis, as high levels of infectious virus have been described in the CNS, spleen, heart, and other organs in these mice [70,71,72,73]. (3) Another strategy has been to infect wild-type mice with SARS-CoV-2 after intratracheal delivery of an adenoviral or adeno-associated viral vector encoding for human ACE2. This method leads to mild to moderately severe disease, and infection is largely restricted to the respiratory tract [38,74]. (4) Lastly, many SARS-CoV-2 variants, including Beta (B.1.351), Gamma, Delta, and Omicron, encode an N501Y mutation in the spike gene that enables binding to murine ACE2 and infection of wild-type laboratory mice. Of these variants, B.1.351 is the most studied: infection of wild-type mice produces moderate weight loss, but does not result in death, and infection appears limited to the respiratory tract [25,75,76,77]. Because of the non-physiologically relevant infection of many extra-pulmonary tissues in K18-hACE2 mice, we will discuss effects in the CNS of mouse-adapted (MA10), B.1.351 variant infection, or AAV-hACE2 transduction followed by SARS-CoV-2 infection.

**Figure 1:**
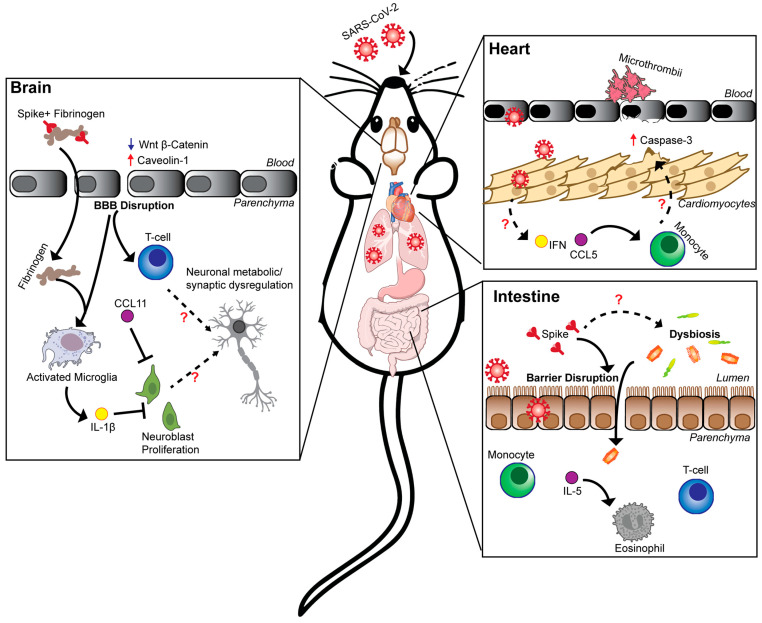
Mechanisms underlying non-respiratory syndromes of PASC in animal models. Intranasal inoculation of NHPs, hamsters, or mice with SARS-CoV-2 have illuminated key mechanisms driving neurological, gastrointestinal, and cardiovascular PASC. Current data suggest that while SARS-CoV-2 does not actively replicate in the brain, alterations in the Wnt–β-catenin pathway and increased caveolin-1 expression promote BBB disruption at acute timepoints. This BBB disruption correlates with increased fibrinogen deposition after peripheral infection, which can activate microglia to produce inflammatory cytokines (such as IL-1β) that inhibit neurogenesis and alter neuronal function. In the intestine, SARS-CoV-2 infection or spike protein in the lumen can promote epithelial barrier disruption. Moreover, infection has been associated with acute dysbiosis, which may drive intestinal inflammation characterized by increased T cell-, monocyte-, and IL-5-dependent eosinophil responses. In the heart, SARS-CoV-2 infection promotes microthrombi formation, which together with direct, yet limited infection of cardiac tissue may initiate type I IFN signaling and CCL5 production to recruit inflammatory monocytes that are hypothesized to mediate tissue damage and cell apoptosis. Solid black arrows indicate mechanisms identified in the literature. Dashed-line arrows indicate hypothesized mechanisms. Some parts of the illustration are derived from NIAID NIH BioArt Source (bioart.niaid.nih.gov/ bioart/# 141, 258, 307, and 415, accessed on 12 December 2024).

Mice may be a good model for studying BBB disruption, as studies with MA10 or B.1.351 in C57BL/6J mice reported evidence of BBB breakdown in the brain stem, hippocampus, and cortex [25,66]. One hypothesis suggests that circulating SARS-CoV-2 spike protein, which has been detected in the sera of PASC patients, binds to endothelial cells and initiates BBB disruption. Indeed, intravenous injection of the S1 subunit of the spike protein resulted in accumulation in the brain parenchyma of mice [78,79]. Fibrinogen also reportedly interacts with the SARS-CoV-2 spike protein, which could cause damage to endothelial barriers [25]. Consistent with this hypothesis, at acute timepoints after MA10 infection of mice, brain microvascular endothelial cells showed reduced expression of genes related to tight junction formation (claudin 5 and occludin) and increased expression of mediators of endocytosis and transcytosis (caveolin 1) [66,67,80]. Activation of the β-Wnt–catenin pathway (an upstream regulator of tight junction proteins) or genetic deletion of caveolin 1 in brain microvascular endothelial cells inhibited BBB disruption after MA10 infection, and these perturbations were associated with decreased microgliosis, astrogliosis, and T cell infiltration [66,80]. In the B.1.351 model, BBB disruption resulted in fibrinogen leakage into the hippocampus, and depletion of fibrinogen prevented microgliosis at acute infection timepoints [25]. BBB disruption also may exacerbate sickness behavior, as behavioral testing of MA10-infected mice at 5 days post-infection showed improvement in memory and motor skills using the novel-object recognition and pole descent tests when BBB disruption was inhibited [66]. Thus, MA10 and B.1.351 mouse models suggest that BBB disruption may initiate or exacerbate neuroinflammation (Figure 1). Although the persistence of BBB disruption in these models at subacute timepoints has not yet been assessed, transient BBB disruption could still contribute to post-acute cognitive deficits. Studies investigating traumatic brain injury demonstrated that acute injury to CNS barriers (including the BBB) initiates neuroinflammation cascades that cause a delayed “secondary injury” and result in cognitive decline [81]. Thus, how BBB disruption in the acute phase of SARS-CoV-2 infection affects long-term cognitive deficits warrants more study. Additionally, the exact cause of BBB disruption after SARS-CoV-2 infection is undetermined.

Mouse models of neuroCOVID have begun to link neuroinflammation with behavioral outcomes. Studies using both MA10 and B.1.351 infection of wild-type mice have described microgliosis, T cell infiltration, and astrogliosis beginning at 5 to 7 days post-infection in the hippocampus and/or brain stem, which persists at least through 28 days post-infection [25,66,67,75,82]. However, these results differ somewhat from data obtained after WA1 infection of hACE2 AAV-transduced mice (AAV-hACE2-WA1 model), which showed microgliosis only in white matter tracts at 7 days post-infection that persisted through 7 weeks post-infection [38]. In both the B.1.351 model and the AAV-hACE2-WA1 model, intranasal SARS-CoV-2 infection resulted in increases in pro-inflammatory cytokines (e.g., IL-1β, IFNβ, CXCL10, IFNγ, IL-6, and CCL11) in the hippocampus and cerebrospinal fluid at 7 days post-infection [38,75,82,83]. However, only a few inflammatory mediators remained elevated at 28 days post-infection in the hippocampus in the B.1.351 model (IL-6) and in the CSF in the AAV model at 7 weeks post-infection (CXCL10, CCL11) [38,82]. Inflammatory cytokines in the brain parenchyma can inhibit crucial neural processes, such as neurogenesis [84]. In both the B.1.351 model and the AAV-hACE2-WA1 model, the number of neuroblasts in the subgranular zone of the dentate gyrus was decreased at 6 to 8 days post-infection, and remained low through 28 days or 7 weeks post infection, respectively [38,75]. One study using the B.1.351 infection model linked the decrease in neurogenesis to neuroinflammation by showing that IL-1β produced by activated microglia in the hippocampus inhibited neural stem cell differentiation into mature neurons at 7 days post-infection [75]. Mature neurons also show evidence of dysfunction at 28 days post-infection, with decreased numbers of excitatory synapses in the dentate gyrus of the hippocampus and reduced neurotransmitter activity after B.1.351 infection [75,83]. At 7 weeks post-infection with AAV-hACE2-WA1, there were decreased numbers of myelinated axons and oligodendrocytes in white matter tracts [38]. Neuropathology was also linked to decreases in recognition memory, as measured using novel-object recognition behavioral testing in B.1.351-infected mice at 28 days post-infection [75]. Collectively, these data indicate that SARS-CoV-2-infected mice develop persistent neuroinflammation, neuronal dysregulation, and memory deficits and thus may be a model to investigate mechanisms driving long-term cognitive deficits.

### Concluding Remarks on NeuroCOVID Animal Models

Substantial progress has been made in developing animal models for neuroCOVID, particularly in rodents (Figure 1). Syrian golden hamsters may allow investigation of the basis for anosmia and peripheral neuropathy, although mechanistic studies may be hampered by the lack of genetic knockout models. Three mouse models of neuroCOVID have been described, with all (MA10, B.1.351, and AAV-hACE2-WA1) showing significant neuroinflammation despite a lack of viral replication in the brain. These models have been useful in studying pathology and inflammation in the hippocampus, brain stem, and white matter tracts of the brain and have associated these findings with long-term alterations to neuronal processes and memory deficits. These different models of neuroCOVID are poised to provide key insights into the underlying mechanisms causing cognitive deficits and potential avenues for therapeutic intervention.

## 3. Gastrointestinal PASC

Many COVID-19 patients present with GI tract symptoms such as nausea, vomiting, diarrhea, abdominal pain, and anorexia during the acute phase of disease. In hospitalized patients, up to 44% report continued GI tract symptoms at 90 days post-infection, and abdominal pain and nausea are still reported at 3 years post-infection by PASC patients [8,85,86]. Current evidence suggests that 29% to 55% of patients infected with SARS-CoV-2 have detectable viral RNA in their stool, which can persist for at least 7 months [86,87,88,89]. Endoscopy studies confirmed that SARS-CoV-2 antigens and viral RNA are detectable in the small and large intestine of PASC patients and that persistence of SARS-CoV-2 correlates with GI tract symptoms [87,89,90]. Changes in the microbiota occur for at least 6 months and correlate with GI PASC (reviewed in [91]). However, it remains unclear how persistent viral RNA in the GI tract and ensuing microbiota perturbations affects chronic disease. Significant barriers have been encountered in developing an animal model with persistent SARS-CoV-2 viral RNA burden in the GI tract.

NHPs are a potential model for GI PASC. Intranasal and intratracheal infection of rhesus macaques led to detectable viral RNA in the stool up to 10 days post-infection [43,88]. SARS-CoV-2 viral RNA or antigen was also detected in rectal swabs or intestinal samples at 3–10 days post infection of rhesus or cynomolgus macaques [92,93,94]. Direct intragastric inoculation of rhesus macaques resulted in SARS-CoV-2 infection of the GI tract, with some variability [95]. Substantive changes in the microbiota composition before and after SARS-CoV-2 infection in NHPs were not detected in one study, although commensal microbes translocated across the colonic epithelium after infection, suggesting at least transient disruption of the gut barrier [88]. Similarly, other NHP studies described evidence of intestinal barrier damage after SARS-CoV-2 infection, including markers of cell death (caspase 3 expression) and intestinal fatty acid-binding protein, the latter of which was elevated through 43 days post-infection [94,95]. An immune response in the GI tract also was observed in NHPs after SARS-CoV-2 infection, as mucosal, SARS-CoV-2-specific, granzyme B^+^ effector T cells were present until at least 43 days post-infection [88,92,94]. Changes in B cell numbers in the GI tract of NHPs after SARS-CoV-2 have also been reported, although some studies show decreases [88,94], whereas others show increases in number [92]. Signs of highly activated macrophages and/or monocytes, changes in Paneth cell numbers, and alterations in inflammatory cytokine levels have been reported in the intestines during acute SARS-CoV-2 infection of NHPs [88,92,94,95]. Thus, NHP models appear to recapitulate some of the characteristics of the GI tract response to SARS-CoV-2 infection in humans, including the presence of viral RNA, damage to the epithelial barrier, and immune responses (Figure 1). Although NHP models are limited by a relative lack of GI disease signs, they may still be useful for investigating mechanisms by which SARS-CoV-2 infection impacts GI tract barrier integrity and immunity.

Few studies have been published describing the effects of SARS-CoV-2 infection on the GI tract in hamsters. Intranasal infection of Syrian golden hamsters resulted in low or no levels of SARS-CoV-2 RNA or antigens in the large intestine [50,96,97,98]. However, even without detectable viral RNA, pathology was observed in the intestine during acute infection, particularly infiltration of eosinophils [96,97]. Accordingly, the eosinophil-activating cytokine IL-5 was elevated in the intestinal samples out to 15 days post-infection, although other inflammatory cytokines investigated (e.g., IL-1β, IL-6, TNF) were unchanged [96]. Taxonomic analysis of the microbiota of hamsters after acute SARS-CoV-2 infection revealed an altered community structure [97]. Furthermore, antibiotic treatment prior to infection with SARS-CoV-2 worsened survival, suggesting that the microbiota might have a role in protection in hamsters [99]. Thus, hamsters may have some dysbiosis and GI tract inflammation after SARS-CoV-2 infection, although the duration and cause of these phenotypes is unknown.

The development of mouse models with evidence of the GI disease reported in humans has been challenging to achieve. Intranasal inoculation of hACE2 knock-in mice, which expresses human ACE2 in the lung, kidney, and small intestine, resulted in detectable viral RNA and antigens in the intestine at acute infection timepoints. Histopathological analysis of the intestine showed evidence of epithelial injury and B cell and neutrophil infiltration [100]. After intranasal inoculation of transgenic K18-hACE2 mice with SARS-CoV-2, low levels of viral RNA were measured in the intestine along with an altered microbiome at 2 to 6 days post-infection [22,71,101]. In wild-type non-transgenic mice, analysis of the small intestine did not reveal histopathological changes in the B.1.351 model [102]. Despite an absence of viral RNA in the intestines of B.1.351-infected mice, changes in the microbiome were present and persisted until 20 days post-infection [22]. Thus, changes in the microbiome may occur independently of productive SARS-CoV-2 infection of the intestine. Indeed, injection of SARS-CoV-2 spike protein directly into the intestine or intraperitoneally was sufficient to cause intestinal barrier disruption in wild-type mice [103,104]. Mouse models may be useful for studying SARS-CoV-2-induced microbiota-mediated changes in the GI tract and the resulting pathological consequences associated with dysbiosis. Whether mouse models develop long-term dysbiosis after SARS-CoV-2 infection remains unknown.

In summary, NHPs appear to be a reasonable model for studying SARS-CoV-2-associated GI tract injury and immunity, with hamsters providing an alternative model, as viral RNA can be found in the GI tract in both models at acute infection timepoints. To our knowledge, only one group has investigated the microbiota of NHPs after SARS-CoV-2 infection, and their study did not detect significant dysbiosis. Further studies are needed to determine if NHPs could be used to investigate the linkage between SARS-CoV-2 infection and dysbiosis. Thus, the development of an animal model that results in persistent viral RNA in the stool or intestine, signs of GI tract disease (vomiting, abdominal pain, and diarrhea), dysbiosis, and persistent histological changes in the intestine remains an important goal.

## 4. Cardiovascular PASC

SARS-CoV-2 infection has a complex relationship with the cardiovascular system. During acute COVID-19 and up to 3 years after recovery, the risk of stroke and heart attack is elevated, even after mild infections [105]. Patients suffering from PASC frequently report cardiovascular symptoms, including chest pain, palpitations, shortness of breath, and light-headedness, many of which persist [8,29,106,107]. Some PASC patients have been diagnosed with new-onset postural tachycardia syndrome, a chronic disease characterized by autonomic dysfunction that often requires lifelong management [108,109,110]. Thus, the burden of cardiovascular PASC is high, although relatively little is known about the underlying pathophysiology. Autopsy studies of COVID-19 patients showed small increases in lymphocytic and monocytic infiltration into the heart, although these were below established criteria for myocarditis [111,112,113]. Two studies of patients that died from COVID-19 found that 43% to 50% of patients had detectable viral RNA in the heart; however, another study found low or undetectable RNA in heart tissue [114,115,116]. While it remains unclear if SARS-CoV-2 can productively infect human heart tissue, myocardial injury after SARS-CoV-2 infection does occur, as indicated by elevated cardiac troponin levels, gene changes associated with a reduction in cardiomyocytes and increased oxidative stress, and the appearance of microthrombi [114,117,118,119].

Relatively few studies have assessed the impact of SARS-CoV-2 on cardiovascular function in animal models. Two NHP studies identified SARS-CoV-2 viral RNA or antigens in ventricular tissue that persisted until 30 days post-infection [120,121]. Histopathological analysis of the heart at 7 days post-infection revealed evidence of myocarditis, with higher levels of macrophages, T cells, and pro-inflammatory cytokines in the heart compared to uninfected control animals [121]. Infected NHPs also had microthrombi in the heart, and blood chemistry indicated ongoing coagulopathy [121,122]. More extensive analyses of heart tissues have been performed in Syrian golden hamsters. At acute timepoints after intranasal inoculation, multiple studies have found SARS-CoV-2 viral RNA or protein in hamster heart tissue, albeit at low levels [123,124,125]. SARS-CoV-2-infected hamsters also had ventricular wall thickening, increased congestion and microthrombi, increased caspase 3 expression, and areas of damaged or necrotic heart tissue [123,125,126]. Transcriptomic analysis of heart tissue showed increases in genes related to the production of reactive oxygen species and decreases in genes related to muscle contraction and oxidative respiration [123,124,125]. Sequencing analyses also demonstrated innate immune system activation, with increases in type I IFN responses and induction of pro-inflammatory cytokines [53,96,124,125]. Thus, SARS-CoV-2 infection of hamsters results in cardiac injury, with the immune response likely playing a pathogenic role (Figure 1).

A limited number of studies in mice have explored whether SARS-CoV-2 causes cardiac dysfunction. Intranasal inoculation of K18-hACE2 transgenic mice resulted in robust SARS-CoV-2 infection of the heart at a level of infection that appeared to be much higher than that observed in humans, making interpretation difficult [71]. In other mouse models (e.g., MA10 or B.1.351) viral RNA is found only when using specific mouse strains, such as the 129/S2 strain [64,102,127]. To overcome this problem, one group created a mouse that expressed human ACE2 under a cardiomyocyte-restricted promoter (*Myh6-Cre*) and then intravenously infected these animals with a SARS-CoV-2 strain (WA1) that cannot bind to mouse ACE2, thus creating a cardiomyocyte-restricted SARS-CoV-2 infection model. The authors found that infection in *Myh6-Cre* hACE2 KI mice was rapidly cleared from the heart (by 7 days post-infection) and led to left ventricular systolic dysfunction, which was driven by CCR2-dependent infiltration of monocytes into the heart [127]. To date, no study has assessed the long-term impact of SARS-CoV-2 infection on cardiac function or integrity in mouse models. Thus, further development of mouse models that can recapitulate the low levels of viral RNA found in heart tissue in humans, yet cause persistent cardiovascular dysfunction are needed.

## 5. Conclusions and Future Perspectives

We have summarized three animal models of extra-pulmonary PASC: NHPs, Syrian golden hamsters, and mice (Table 1). While murine models are useful for studying neuroCOVID, they provide less insight into the GI tract or cardiac disease seen in humans. Intranasal infection of hamsters is an effective model for some features of neuroCOVID, particularly anosmia. Moreover, hamsters may be a promising model for some GI tract and cardiovascular sequelae as well, considering that viral RNA and signs of tissue pathology can be detected in the heart and intestine. NHP models recapitulate several extra-pulmonary sequalae, especially GI tract and neuronal pathology; however, NHP studies are costly, and it is challenging to power studies to detect smaller phenotypes. One caveat is that for all the organ systems described above (neurological, cardiovascular, gastrointestinal), most of the studies were performed at acute or subacute (30 days post-infection or less) timepoints. As PASC can persist for at least 3 years, it will be essential to assess the durability of these sequalae at later timepoints in each animal model.

We focused on animal models of neurological, cardiovascular, and gastrointestinal sequelae, as these systems had the largest number of published studies available. However, there are several other major organ systems affected by PASC that have few or no animal studies. Musculoskeletal weakness and post-exertional malaise are commonly reported symptoms by PASC patients and are associated with alterations in muscle structure and mitochondrial dysfunction [2,8,128]. To date, no direct assessment of muscle tissue has been performed in any animal model. However, one study using Syrian golden hamsters found that intranasal SARS-CoV-2 inoculation led to a myeloid and IL-1β inflammatory response in the bone that promoted activation of osteoclasts, resulting in reduction in trabecular bone volume from 4 days post-infection and continuing until at least 60 days post-infection [129]. Liver, pancreas, and kidney injury have also been reported in COVID-19 patients. Two studies in NHPs found evidence of liver and kidney injury and changes in metabolites in these organs, which was accompanied by hyperglycemia [130,131]. Hormonal changes also have been reported in PASC patients [16,132], and one NHP study found evidence of tissue damage in the testes during acute SARS-CoV-2 infection that was associated with hormonal fluctuations [133]. One consideration in studying hormonal changes in PASC is that the female sex is strongly associated with increased risk of PASC in humans and that immune responses (e.g., TGF-β expression and monocyte profiles) in male and female PASC patients vary [134,135,136,137,138]. While several studies have reported increased susceptibility and disease severity in male mice and hamsters during acute SARS-CoV-2 infection [83,139,140], few have investigated the impact of sex on PASC. One report in hamsters observed that neuronal changes in the brain were greater in females, whereas two studies in mice showed little impact of sex on neuroCOVID [58,75,83]. More consideration of the effect of sex on different features of PASC, as well as novel animal models for liver, kidney, and reproductive tract injury and post-exertional malaise associated with SARS-CoV-2 infection and PASC, are needed.

Animal models present the potential to test many hypotheses associated with the pathogenesis of PASC, advance development of therapeutics, and inform ongoing clinical trials. (1) Persistent viral RNA hypothesis. Both NHP and hamster models have extrapulmonary SARS-CoV-2 RNA and could provide an opportunity to investigate how modulating viral RNA levels and/or persistence (for example with antiviral treatment) impacts sequelae. Understanding these interactions will be important for advancing treatments for PASC, as ongoing clinical trials for PASC with antiviral drugs have had mixed efficacy in preliminary results [141,142]. (2) Dysregulated immunity hypothesis. A common feature of neurological, gastrointestinal, and cardiovascular sequelae in all animal models discussed here is evidence of immune activation (Table 1). While mouse models have identified IL-1β, CCL11, and microglia as drivers of acute neuroCOVID, more research is needed to determine if these effects persist in the long term [38,66,75]. Further studies are needed to address the role of adaptive immunity in neuroCOVID and whether and how innate or adaptive immunity drives GI or cardiovascular sequelae. Ongoing clinical trials are assessing the impact of metformin, an anti-inflammatory and blood sugar-reducing drug, on long-term PASC sequelae, as treatment with metformin at acute timepoints lowered the risk of PASC [143]. Elucidation of the specific immune mechanisms driving PASC in animal models may help identify more targeted anti-inflammatory therapeutics.

Several other major hypotheses of the pathogenesis of PASC still need better animal models for exploring mechanisms and evaluating therapeutics. (3) Dysbiosis hypothesis. NHPs, hamsters, and mice had evidence of dysbiosis after SARS-CoV-2 infection. One group found that virus-induced inflammation in mice inhibited intestinal amino acid absorption, which resulted in decreased systemic serotonin (a feature of PASC), ultimately causing neurocognitive effects [132]. Further work investigating the gut–brain axis after SARS-CoV-2 infection could illuminate additional pathways driving PASC. (4) Coagulopathy hypothesis. Several groups have investigated dysregulation of the coagulation cascade during SARS-CoV-2 infection. Infection of wild-type mice with MA10 results in coagulopathy and fibrin deposition in the lung at acute timepoints that is partly caspase 11-dependent and can be inhibited by treatment with the anti-thrombin/complement inhibitor serpin 1 [65,144,145]. Another study demonstrated that direct binding of the spike protein to fibrinogen leads to fibrin clot formation in the lung and brain after B.1.351 infection of wild-type mice, which increases inflammation and adverse outcomes [25]. While mouse models have helped our understanding of coagulopathy during acute SARS-CoV-2 infection, further studies are needed to directly link it to PASC. Alternatively, (5) dysregulation of the hypothalamic–pituitary–adrenal (HPA) axis, which can cause cognitive difficulty, fatigue, and other mood disorders, has been proposed to underlie PASC in many patients. Decreased cortisol levels are a strong predictor of development and maintenance of PASC [16,146,147,148,149]. To the best of our knowledge, changes in cortisol levels and HPA axis function have not yet been directly demonstrated in animal models. Preliminary clinical trials suggest that treatment with corticosteroids may improve symptoms in PASC patients, and larger trials are underway to provide further evidence [150,151]. Developing an animal model for cortisol insufficiency after SARS-CoV-2 could help elucidate how the HPA axis contributes to features of PASC and the mechanism of action behind successful interventions. Animal models also may be useful for understanding why decreased cortisol production (and other factors such as sex) are risk factors for PASC.

Animal models have already advanced our understanding of the mechanisms driving extrapulmonary pathology during the acute and subacute phases of COVID-19 (Figure 1). However, these models have only begun to explore persistent extrapulmonary pathology and the underlying mechanisms driving PASC. Further research will hopefully provide unifying hypotheses for the diverse sequelae of PASC and identify candidates for future therapeutics.

## Figures and Tables

**Table 1:** Comparison of animal models of PASC.

	NHP	Hamster	Mouse				
			MA10	B.1.351	AAV-hACE2	K18-hACE2	*My6h*Cre x hACE2
Neurological							
Viral RNA	Conflicting data	Yes, in OB	No	No	No	Yes	
Neuroinflammation: microgliosis, T cell infiltration, cytokine production	Yes	Yes	Yes	Yes	Yes	Yes	
BBB disruption		Yes	Yes	Yes			
Neuronal dysfunction		Yes		Yes	Yes		
Behavioral changes		Yes	Yes	Yes			
Gastrointestinal							
Viral RNA	Yes	Conflicting data		No		Yes	
Inflammation	Yes	Yes				Yes	
Tissue damage	Yes		No				
Dysbiosis	Conflicting data	Yes		Yes		Yes	
GI signs (diarrhea, etc.)	Not observed	Not observed					
Cardiovascular							
Viral RNA	Yes	Yes	No	In 129/S mice only		Yes	Yes
Inflammation	Yes	Yes					Yes
Tissue damage		Yes	No				Yes
Coagulopathy	Yes		Yes	Yes			
Functional changes							Yes
Sex-specific symptoms							
Acute disease severity	Some reports, but *N* is too small	Yes	Yes	Yes		Yes	
Post-acute neuronal sequelae		Yes		No			

Empty space, Unknown for that animal model.
